# Ten‐year update on the European Association of Urology Robotic Section (ERUS) fellowship training for robot‐assisted radical prostatectomy

**DOI:** 10.1002/bco2.70227

**Published:** 2026-07-08

**Authors:** Marco Paciotti, Christian Wagner, Nicola Frego, Edoardo Beatrici, Angelo Mottaran, Carlo Andrea Bravi, Federico Piramide, Maria Peraire Lores, Luca Sarchi, Adele Piro, Rui Farinha, Gabriele Sorce, Eleonora Balestrazzi, Luigi Nocera, Claudia Collà Ruvolo, Silvia Rebuffo, Nikolaos Liakos, Paolo Dell'Oglio, Mike Wenzel, Marcio Covas Moschovas, Filippo Turri, Fabrizio Di Maida, Edward Lambert, Christoph Wurnschimmel, Ruben De Groote, Ben van Cleynenbreugel, Alessandro Larcher, Iulia Andras, Nicolò Maria Buffi, Henk G. van der Poel, Alberto Breda, Alexandre Mottrie, Anthony G. Gallagher, ERUS Working Group on Education and Junior ERUS/YAU Working Group on Robot‐assisted Surgery

**Affiliations:** ^1^ Department of Biochemical Science Humanitas University Milan Italy; ^2^ Department of Urology Humanitas Clinical and Research Institute IRCCS Milan Italy; ^3^ Department of Urology Urologic Oncology and Robotic Surgery, St. Antonius‐Hospital Gronau Germany; ^4^ Department of Urology Humanitas Mater Domini Varese Italy; ^5^ ORSI Academy Melle Belgium; ^6^ Department of Urology Onze‐Lieve‐Vrouwziekenhuis Hospital Aalst Belgium; ^7^ Division of Urology IRCCS University Hospital of Bologna Bologna Italy; ^8^ Department of Urology Northampton General Hospital Northampton UK; ^9^ Division of Urology, Department of Oncology San Luigi Gonzaga Hospital, University of Turin Turin Italy; ^10^ Servicio de Urologia del Hospital Universitario Joan XXIII de Tarragona Tarragona Spain; ^11^ Department of Urology IEO European Institute of Oncology, IRCCS Milan Italy; ^12^ Department of Urology Dolo Hospital Dolo Italy; ^13^ Department of Urology, Division of Oncology Urological Research Institute, IRCCS Ospedale San Raffaele Milan Italy; ^14^ Vita‐Salute San Raffaele University Milan Italy; ^15^ Department of Neurosciences, Science of Reproduction and Odontostomatology University of Naples Federico II Naples Italy; ^16^ IRCCS Ospedale Policlinico S. Martino Genoa Italy; ^17^ Department of Urology, Faculty of Medicine Medical Centre of the University of Freiburg Freiburg Germany; ^18^ ASST Grande Ospedale Metropolitano Niguarda Urology Department Milan Italy; ^19^ Department of Urology Goethe University Hospital Frankfurt Frankfurt am Main Germany; ^20^ AdventHealth Global Robotics Institute Celebration Florida USA; ^21^ A. Gemelli University Hospital Foundation IRCCS Rome Italy; ^22^ Department of Experimental and Clinical Medicine University of Florence ‐ Unit of Oncologic Minimally‐Invasive Urology and Andrology, Careggi Hospital Florence Italy; ^23^ Department of Urology Luzerner Kantonsspital Lucerne Switzerland; ^24^ Department of Development and Regeneration KU Leuven Louvain Belgium; ^25^ Department of Urology Iuliu Hatieganu University of Medicine and Pharmacy Cluj‐Napoca Romania; ^26^ Department of Urology Netherlands Cancer Institute ‐ Antoni van Leeuwenhoek Hospital Amsterdam The Netherlands; ^27^ Department of Urology Fundació Puigvert Barcelona Spain; ^28^ Department of Surgery Autonomous University of Barcelona Barcelona Spain; ^29^ School of Medicine, Faculty of Life and Health Sciences Ulster University Belfast UK

**Keywords:** curriculum, fellowship training, patient safety, robot‐assisted radical prostatectomy, robotic surgery, surgical education

## Abstract

**Objective:**

The aim of this paper is to evaluate fellowship outcomes 10 years after implementation of the European Association of Urology Robotic Section (ERUS) structured curriculum for robot‐assisted radical prostatectomy (RARP), with a focus on completion rates and reasons for non‐completion.

**Subjects and methods:**

Data were obtained from institutional records and a trainee survey. The primary outcome was fellowship completion (i.e., Certificate of Excellence achievement). Secondary outcomes included reasons for non‐completion and satisfaction. Completion rates were analysed annually, with trends assessed using the Cochran–Armitage test and log‐linear regression for the Estimated Annual Percentage Change (EAPC). Comparisons before and after introduction of a procedural diary (2023) and between pandemic and non‐pandemic years used Fisher's Exact Test.

**Results:**

Among 126 fellows, a total of 42 (33%) completed the fellowship by achieving the Certificate of Excellence. The trainee survey achieved a response rate of 77%, supporting the representativeness of the collected data. The main barriers to fellowship completion included limited console access (49%), insufficient programme duration (20%), logistical difficulties (20%) and COVID‐19‐related disruptions (11%). Despite these limitations, overall satisfaction with the fellowship was high (83%), with particularly strong approval of the ORSI hands‐on training week (100%). Completion rates demonstrated a progressive increase over time, rising from 20% in 2018 to 52% in 2023. The Cochran–Armitage test confirmed a statistically significant upward trend in completion rates over the study period (*p* < 0.001), while log‐linear regression analysis showed a numerical but non‐significant EAPC of 13% (95% CI –0.6 to 28.6). Although 2023 represented the highest observed completion rate, this peak was not significantly different from previous years (OR 2.63, 95% CI 0.91–7.63).

**Conclusions:**

The RARP ERUS Fellowship remains a benchmark in robotic training, but unsatisfactory completion rates highlight the need for improvement. Recent reforms, including the procedural diary, show promise and warrant expansion.

## 1 INTRODUCTION

In the last two decades, robotic urologic surgery has transformed the way complex procedures are performed. One of the key elements for the successful implementation of robotic surgery is, however, comprehensive and structured training for surgeons. Indeed, along with the dissemination of robot‐assisted surgery, multiple scientific societies and training bodies expressed the imperative for structured training programmes in robotic surgery.^1^, ^2^, ^3^ The development of a standardized training curriculum, including training of trainers, assessment and certifications of the trainees, clearly emerged as a priority in surveys conducted among international experts at the 2012 European Association of Urology Robotic Section (ERUS) Congress and the 2013 European Association of Urology (EAU) meeting.^3^ As a consequence, ERUS developed a structured training programme focused on robot‐assisted radical prostatectomy (RARP).^3^, ^4^ This curriculum represented a pioneering step in the field and has since been recognized as a reference model for the development of structured training programmes in robotic surgery. It was developed according to a comprehensive multi‐step model, beginning with online theoretical modules, followed by simulation‐based training and culminating in a clinical fellowship. During the fellowship stage, participants were trained to progressively perform the surgical steps of the procedure, from basic to advanced tasks, eventually performing the entire RARP procedure.^5^ At the end of the programme, trainees' ability to perform RARP was assessed through a blinded review of the video‐recorded full procedure, performed independently yet under the supervision of their mentors.^3^, ^4^, ^5^ After the initial pilot study, several studies have confirmed the validity of the fellowship but also highlighted programme issues.^6^, ^7^, ^8^ In particular, the data from the 5‐year update survey suggested a low completion rate.^8^ In the following years, however, the ERUS fellowship programme continued to successfully train dozens of robotic surgeons across Europe. Nonetheless, given the centrality of completion rate as a potential indicator of both training effectiveness and real‐world feasibility, a critical reassessment of programme outcomes was warranted.

Herein, we present an update 10 years after the development of the curriculum. Specifically, we reviewed the data on fellowship completion and conducted an in‐person, telephone follow‐up survey of the trainees to investigate any reasons for non‐completion of the fellowship.

## 2 METHODS

### 2.1 Study design, data source and survey

This is a retrospective cohort study with a cross‐sectional survey component. It was conducted using data retrieved from a prospectively maintained institutional database at ORSI Academy. All trainees officially enrolled in the RARP ERUS fellowship between January 2018 and December 2023 were included in the analysis. Fellows who participated in the ORSI Academy RARP course as a standalone activity, without being part of the RARP ERUS fellowship, were excluded.

To gather additional qualitative and subjective data, a structured survey was conducted among all fellows who entered the programme from 2018 to 2022. Data on the 2022 cohort were collated in 2023. The survey aimed to assess satisfaction with the fellowship, self‐perceived surgical autonomy at completion, and reasons for non‐completion where applicable. Data were collected via standardized telephone interviews using a predefined questionnaire (supplementary material), and results were analysed descriptively.

In early 2023, the fellowship programme introduced a structured procedural diary, an online platform through which trainees are required to upload video recordings of each modular phase of the RARP procedure as they progress through their training. This system facilitates both self‐assessment and structured mentor evaluation, using validated binary metrics for each procedural step.^9^

### 2.2 Study endpoints and other variables

The primary endpoint of this study was the fellowship completion rate. Completion rate was defined as the proportion of RARP ERUS fellows who were awarded the final Certificate of Excellence upon fulfilment of all fellowship requirements. These included the independent and competent execution of a complete RARP case at a certified ERUS host centre, as well as the submission of a full‐case video for evaluation. Only fellows who met the established objectively assessed performance standards were considered to have successfully completed the programme.

Secondary outcomes included trainee‐reported satisfaction with the fellowship, the level of surgical autonomy achieved by the end of the training, and the reasons for non‐completion in cases where the certificate was not awarded.

### 2.3 Statistical analysis

Fellowship completion rates were reported as percentages. A Cochran–Armitage test was applied to assess the presence of a linear trend in completion rates over time. In addition, a log‐linear regression model was used to calculate the Estimated Annual Percentage Change (EAPC) in completion rates with corresponding 95% confidence intervals (COIs). This exploratory analysis aimed to quantify the average yearly change in performance and evaluate whether the observed trend was statistically significant. Survey responses were summarized as percentages.

### 2.4 Procedural diary and comparative analysis

To evaluate the potential educational impact of the procedural diary, implemented in early 2023, completion rates of fellows trained prior to its introduction (2018–2022) were compared with those of the 2023 cohort using Fisher's Exact Test. This preliminary comparative analysis aimed to determine whether the procedural diary was associated with improved training outcomes and higher completion rates.

### 2.5 Sensitivity analysis

As part of the survey, trainees were given the opportunity to indicate whether disruption of the fellowship programme due to the COVID‐19 pandemic was a contributing factor to their failure to complete the curriculum. To better account for the potential confounding impact of the pandemic on training progression and fellowship completion, a sensitivity analysis was conducted. Specifically, completion rates for fellows enrolled during the pandemic years (2020–2021) were calculated and compared to those from non‐pandemic years using Fisher's Exact Test.

This analysis allowed us to assess whether the overall completion rate was disproportionately affected by disruptions in clinical activity, travel restrictions or reduced surgical exposure during those years.

## 3 RESULTS

### 3.1 Completion rate and reason for non‐completion

A total of 208 trainees attended the RARP course at ORSI Academy between January 2018 and December 2023. Of these, 82 (39%) participated in the course as a standalone option and were therefore excluded from further analysis. The remaining 126 trainees (61%) attended the course as part of the RARP ERUS fellowship and were included in the analysis.

**FIGURE 1:**
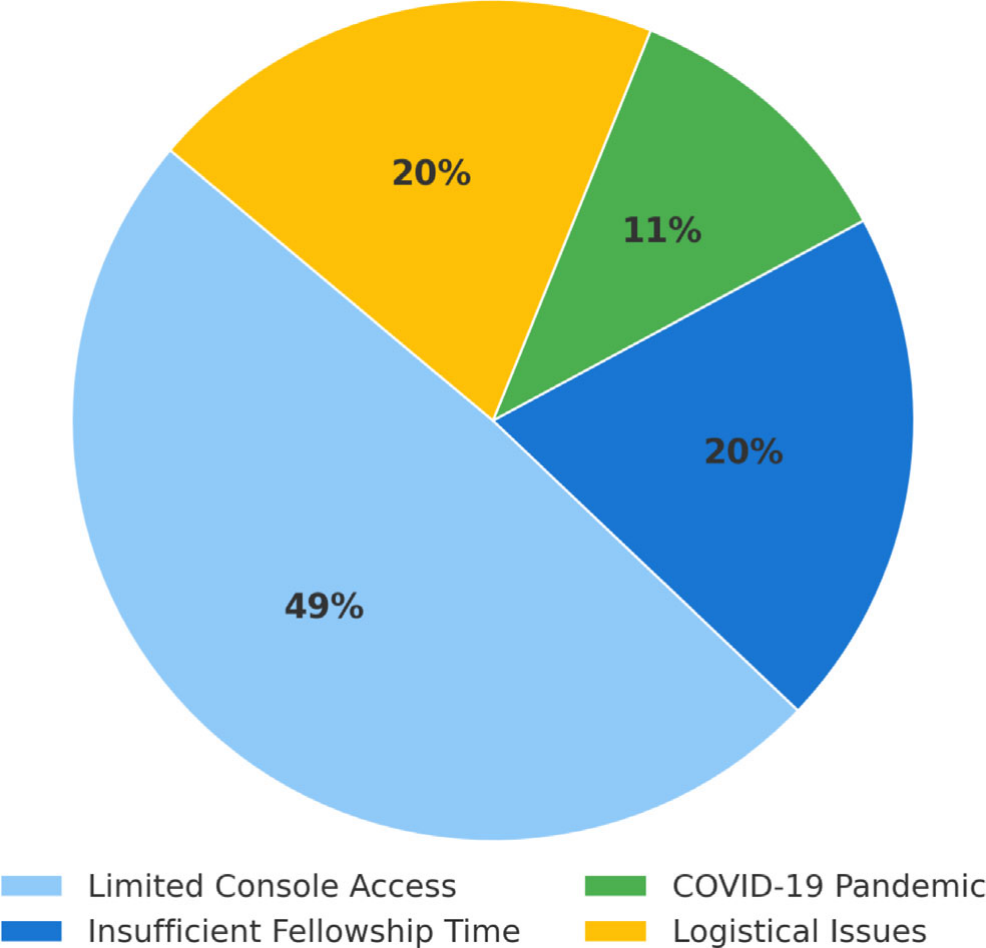
Reasons for fellowship non‐completion as reported in the survey. Responses were grouped into four main categories: (1) limited console access: included insufficient access to the robotic console or restricted surgical autonomy at the host centre. Some fellows reported not being allowed to perform the entire procedure, particularly the nerve‐sparing phase. (2) Insufficient fellowship time: included cases where fellows felt that 6 months were not enough to complete the programme. This applied to those who needed additional time to perform a full case and those who completed the fellowship without having done so independently. (3) Logistical issues: included lack of clarity regarding the video submission process, missed deadlines or failure to record and submit the surgical video. In some instances, fellows reported having completed a full case but not recording it, while one fellow noted having submitted a video that was never assessed. (4) COVID‐19 pandemic: included disruptions to the fellowship pathway due to pandemic‐related restrictions or institutional limitations.

Overall, 42 out of 126 fellows (33%) successfully achieved the Certificate of Excellence. An online and in‐person (telephone) survey was conducted among fellows who began the fellowship between 2018 and 2022 to explore reasons for non‐completion. The response rate was 77%, which is considerably higher than that of similar surveys conducted in the past.^8^ The completion rate among respondents was 34%, making this sample representative of the overall population. The fellows' responses were analysed and grouped according to a consensus reached by the interviewers. The main causes identified by the interviewed fellows for not completing the fellowship (i.e., not achieving the Certificate of Excellence) included limited access to the robotic console (49%), insufficient duration of the fellowship (20%), logistical difficulties in receiving instructions, recording and/or submitting the full‐case video (20%) and disruptions to the fellowship pathway caused by the COVID‐19 pandemic (11%). The main reasons contributing to the unsatisfactory completion rate are summarized in Figure 1, together with a detailed breakdown of responses.

### 3.2 Secondary outcomes

Trainee‐reported satisfaction with the fellowship was high, with 83% of respondents expressing satisfaction or high satisfaction with the overall training. Among fellows who did not complete the fellowship, nearly 1 in 10 (9%) self‐reported being able to independently perform the entire RARP procedure and had done so within 1 year of starting the fellowship. The vast majority (68%) reported having performed most or all individual phases of the surgery during their fellowship, even if they had not independently completed a full case. Notably, 100% of surveyed trainees expressed satisfaction or high satisfaction with the one‐week hands‐on training at ORSI Academy, consistently identifying it as a key strength of the programme.

### 3.3 Completion rate per year and trend analyses

**FIGURE 2:**
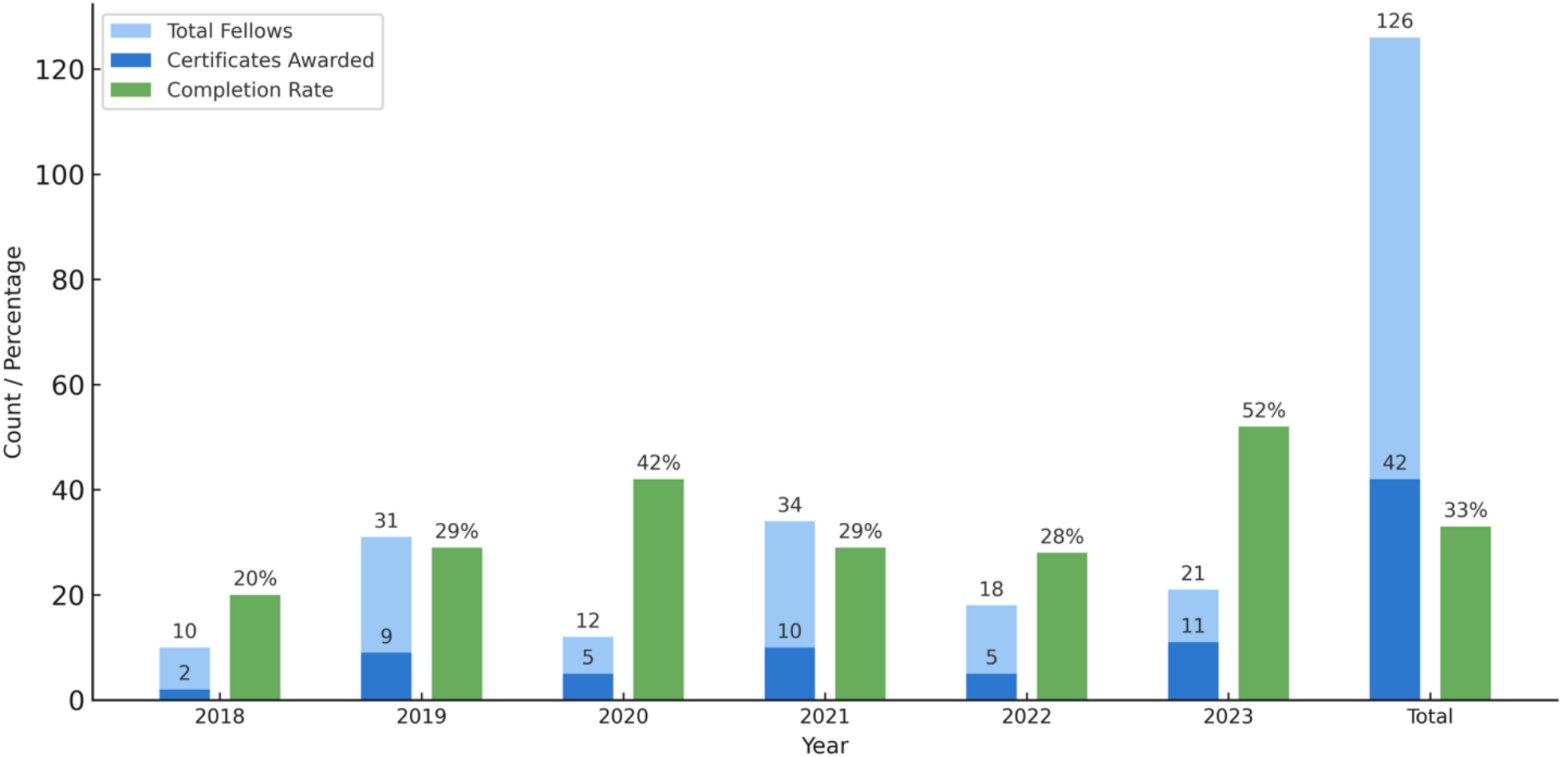
Performance of the robot‐assisted radical prostatectomy (RARP) European Association of Urology Robotic Section (ERUS) fellowship from 2018 to 2023, showing the total number of fellows, those awarded the Certificate of Excellence and the programme completion rate.

Annual analysis of the completion rate revealed values of 20%, 29%, 42%, 29%, 28% and 52% for the years 2018, 2019, 2020, 2021, 2022 and 2023, respectively (Figure 2). Following the survey, video submissions were accepted and evaluated for those trainees who had reported clear logistical issues as the sole reason for not completing the fellowship. As a result, five additional participants were allowed to submit their full‐case videos, performed at their respective ERUS host centres and received the Certificate of Excellence following a positive evaluation. This brought the overall completion rate to 37% (47 out of 126 fellows). A Cochran–Armitage test confirmed a significant upward trend over time (*Z* = 3.71, *p* < 0.001). In contrast, the log‐linear regression model estimated an average increase of 13.1% per year (EAPC), although this did not reach statistical significance (95% CI: −0.6 to 28.6, *p* = 0.135).

### 3.4 Additional analyses

No statistically significant difference in completion rates was observed between non‐pandemic and pandemic years according to Fisher's Exact Test (34% vs 33%, respectively; Odds Ratio [OR]: 0.95; 95% CI: 0.40–2.22; *p* = 0.988). Eleven out of 21 ERUS fellows enrolled in 2023—the final year included in the analysis—submitted a video and successfully obtained the Certificate of Excellence following a positive assessment. This corresponded to a completion rate of 52% in 2023. However, despite this encouraging trend over time, the increase compared to previous years did not reach statistical significance (52% vs. 30%, OR: 2.63; 95% CI: 0.91–7.63; *p* = 0.073).

## 4 DISCUSSION

To the best of our knowledge, the ERUS Fellowship in RARP was the first structured, international, specialist society–based training curriculum specifically designed to ensure safe and effective learning in robotic urologic surgery.^6^ Ten years after its inception, we aimed to assess the status of the programme, seeking to investigate both its strengths and areas requiring improvement. Our analysis revealed that while the programme consistently received high satisfaction ratings and enabled many trainees to perform most procedural steps, it is also characterized by a relatively low overall completion rate, with only 37% of enrolled fellows ultimately receiving the Certificate of Excellence. Importantly, all fellows included in the analysis had sufficient follow‐up time to complete the programme, including those enrolled in 2023, minimizing the risk of underestimating completion rates due to delayed certification.

Over the past decade, the fellowship and its affiliated host centres have played a pivotal role in developing a generation of robotic surgeons. In particular, the one‐week intensive hands‐on training course at ORSI Academy emerged as a key asset, offering a safe and comprehensive initiation into robotic surgery. This module provided a solid theoretical and technical foundation upon which clinical practice could be built. Indeed, the hands‐on training week was consistently praised by trainees interviewed in our survey.

Despite its ability to train a high number of surgeons, the programme showed several critical limitations that must be addressed. Our study clearly demonstrated that the completion rate remains unsatisfactory, largely due to challenges encountered during the host centre phase. These included limited access to the robotic console and variability in training quality and intensity. To a lesser extent, logistical difficulties in completing the required video submission for certification were also reported. Of note, 9% of non‐completing fellows reported having independently performed a full RARP within 1 year, raising the question of whether non‐completion may, in some cases, reflect barriers in certification logistics rather than inadequate training. In line with this, up to 20% of survey respondents cited logistical issues as a reason for not obtaining the Certificate of Excellence. The objective of enabling young surgeons to achieve full procedural autonomy within 6 months may be overly ambitious. Notably, 20% of non‐completing fellows cited insufficient fellowship duration as a key limitation, suggesting that while the training was perceived as high quality, additional time was required to attain full proficiency. This finding underscores the importance of selecting high‐volume, well‐equipped, and committed host centres, staffed by experienced trainers who can offer consistent console access and effective mentorship. Despite stringent selection criteria established since the beginning of the fellowship, our results demonstrate that many pre‐selected high‐volume institutions are still unable to deliver adequate training. Nearly half of the fellows reported insufficient console access, and one in five indicated that overall exposure during the 6‐month programme was inadequate. Taken together, these issues emerged as the main barriers to achieving surgical autonomy. The integration of standardized procedural metrics, train‐the‐trainer courses and dual‐console systems into the curriculum could strengthen the training framework and help ensure meaningful operative exposure for all fellows.^10^ However, these shortcomings highlight the need for stricter monitoring of host centre performance and the implementation of mechanisms to guarantee adequate surgical training. Ultimately, addressing these deficiencies will require enforceable standards and regular auditing of host centres. In this context, also, the use of the Certificate of Excellence as the primary marker of training success warrants further consideration. While it represents a structured and standardized endpoint, it may not fully capture the complexity of surgical competence. The adoption of validated objective performance metrics is likely to represent an important step forward in the assessment of surgical training. Integrating alternative outcome measures, such as independent case volume or perioperative outcomes, remains a complex challenge, but could ultimately provide a more comprehensive evaluation of training effectiveness. In parallel, ongoing technological developments may further influence both the structure and assessment of surgical training. While the increasing diversification of robotic platforms may introduce additional complexity, emerging solutions such as tele‐mentoring may help mitigate these challenges by enabling remote supervision and more flexible training models. Moreover, tele‐mentoring could facilitate a more continuous and objective assessment of trainee performance, complementing existing evaluation frameworks. In this context, technological innovation may ultimately contribute to improved organization of the fellowship and potentially support higher completion rates.

Although the fellowship represents one of the most advanced training curricula in robotic surgery, its early development lacked a strong scientific foundation.^11^ Specific shortcomings included reliance on non‐validated assessment tools (e.g., VR simulators or subjective Likert‐scale evaluations), arbitrary performance benchmarks and insufficient documentation of feedback provided to trainees.^12^

To address these gaps, ERUS has increasingly embraced evidence‐based training principles. The fellowship has recently undergone a significant transformation, grounded in the adoption of Proficiency‐Based Progression (PBP) methodology.^13^, ^14^ This approach, supported by growing scientific evidence, defines explicit performance metrics derived from detailed procedural deconstruction. Trainees must meet these objectively measured standards before progressing to clinical practice, ensuring both safety and skill level. Studies have shown that PBP training can reduce intraoperative errors by approximately 60% compared to conventional methods.^15^ Evidence has also shown that all PBP trainees can achieve the proficiency benchmark and do so significantly faster than those trained using conventional methods.^16^ The cornerstone of this evolution was the formal development and validation of RARP‐specific metrics by the ERUS Scientific and Educational Working Groups.^9^

This commitment to scientifically rigorous training has led ERUS to expand metric‐based curricula to other procedures, most notably robotic‐assisted partial nephrectomy, reinforcing the organization's role as a leader in surgical education.^17^, ^18^, ^19^, ^20^, ^21^, ^22^ Moreover, in order to further enhance quality and consistency, ERUS has implemented systematic quality control measures for host centres, including regular reassessments and ongoing performance monitoring. These interventions are designed to prevent the designation and associated financial support of centres that are not equipped to provide high‐quality training.

A final, but critical, innovation has been the introduction of a procedural diary. This tool facilitates both self‐monitoring and structured assessment, tracking fellows' progress through the modular components of RARP. Piloted in early 2023, the diary, together with the aforementioned reforms, has shown promising results. Indeed, 2023 recorded the highest completion compared to previous years, reaching 52%. While this improvement has not yet reached statistical significance compared to the median of the previous 4 years, it suggests a positive impact of the newly implemented measures. Notably, while the Cochran–Armitage test demonstrated a statistically significant trend over time (*p* < 0.001), the EAPC derived from log‐linear regression was not statistically significant (*p* = 0.135). This apparent discrepancy likely reflects the different statistical properties of the two methods: the Cochran–Armitage test is more sensitive to consistent monotonic trends across ordered groups, whereas the EAPC estimates the magnitude of change over time and is more influenced by variability and sample size. Taken together, these findings suggest the presence of a statistically detectable trend, although the magnitude of change remains modest.

This study provides a critical snapshot of one of the most prominent training programmes in robotic surgery worldwide. It includes data from over 100 ERUS trainees and integrates a detailed survey with a notably high response rate of 77%, enhancing the credibility of our findings.

Nonetheless, some limitations must be acknowledged. First, the sample size, while relatively large for this type of study, remains limited, potentially affecting the power of subgroup analyses and the generalizability of certain conclusions. Second, survey‐based results were self‐reported by fellows, which may introduce recall or response bias. Furthermore, although trainee satisfaction ratings were considered in our evaluation, it is important to note that several studies have questioned the correlation between satisfaction scores and actual training quality or outcomes.^23^

## 5 CONCLUSION

While the RARP ERUS Fellowship remains a pioneering and influential model in robotic surgical education and has already served as a blueprint for other society‐based curricula, our findings highlight its unsatisfactory completion rate and the need for continued refinement. By adopting scientifically validated methodologies such as PBP, strengthening quality assurance across host centres, and promoting comprehensive mentorship and structured evaluation, the programme is well‐positioned to enhance training outcomes and reaffirm its role as a global leader in robotic surgery education.

## AUTHOR CONTRIBUTIONS

*Conceptualization*: Alberto Breda, Anthony G. Gallagher, Alexandre Mottrie, Marco Paciotti, Henk G. van der Poel and Christian Wagner. *Methodology*: Anthony G. Gallagher, Alexandre Mottrie and Marco Paciotti. *Software*: Not applicable. *Validation*: All authors. *Formal analysis*: Marco Paciotti. *Investigation*: Eleonora Balestrazzi, Edoardo Beatrici, Carlo Andrea Bravi, Claudia Collà Ruvolo, Rui Farinha, Nicola Frego, Angelo Mottaran, Luigi Nocera, Marco Paciotti, Maria Peraire Lores, Federico Piramide, Adele Piro, Silvia Rebuffo, Luca Sarchi and Gabriele Sorce. *Resources*: Not applicable. *Data curation*: Anthony G. Gallagher and Marco Paciotti. *Writing—original draft*: Marco Paciotti. *Writing—review and editing*: All authors. *Visualization*: Not applicable. *Supervision*: Anthony G. Gallagher and Alexandre Mottrie. *Project administration*: Not applicable. *Funding acquisition*: Not applicable.

## CONFLICT OF INTEREST STATEMENT

None.
